# Autosomal dominant chronic mucocutaneous candidiasis with STAT1 mutation can be associated with chronic active hepatitis: A case report

**DOI:** 10.3389/fped.2022.990729

**Published:** 2023-01-06

**Authors:** Lingling Liu, Yuan Huang, Yi Liao, Sainan Shu

**Affiliations:** Department of Pediatrics, Tongji Hospital, Tongji Medical College, Huazhong University of Science and Technology, Wuhan, China

**Keywords:** chronic mucocutaneous candidiasis, GOF-STAT1 mutation, liver dysfunction, fungal infections, pediatric

## Abstract

**Background:**

Chronic mucocutaneous candidiasis (CMC) is a heterogeneous primary immunodeficiency disease characterized by chronic or recurrent *Candida* infections of the skin, nails, and mucosa and is mostly associated with STAT1 gain-of-function (GOF) mutation (GOF-STAT1 mutation).

**Case presentation:**

A two-year-old girl was presented with recurrent liver dysfunction, oral candidiasis, acute bronchial pneumonia, and cytomegalovirus infection. Even after a series of treatments, including antifungal voriconazole, nystatin treatment for oral *Candida*, antibiotics against bacterial infection, and bicyclol to protect the liver, the child still exhibited signs of splenomegaly. Although we performed relevant etiological tests on the child and conducted histopathology and electron microscopic examination of the liver, we could not explain the clinical symptoms. So, a genetic test was conducted to clarify the diagnosis. Since the child suffered recurrent fungal infections, we speculated that she had combined immunodeficiency. Therefore we performed high-precision clinical display PLUS detection and found that the transcription factor STAT1 had a heterozygous GOF mutation (*p*. R274W) in its coiled-coil domain.

**Conclusion:**

The clinical manifestations of chronic mucocutaneous candidiasis caused by GOF-STAT1 mutations are complex and range from mild local fungal infections to severe systemic diseases and are sometimes fatal. Clinicians need to be aware of the possibility of this disease in children with recurrent fungal infections for early diagnosis and treatment.

## Introduction

Chronic mucocutaneous candidiasis (CMC) is a heterogeneous primary immunodeficiency disease which is characterized by recurrent or chronic infections affecting the skin, nails, and oral and genital mucosae caused by Candida species (1). CMC disease (CMCD) refers to primary immunodeficiency without any obvious clinical signs. CMC can be classified as CMC syndrome or CMC disease based on its clinical manifestations and the mutations involved. This definition, however, is not exact. On the other hand, patients with syndromic CMC have various clinical and infectious symptoms in addition to CMC. Syndromic CMC involves hyper-IgE syndrome (HIES), autoimmune polyendocrinopathy-candidiasis-ectodermal dystrophy (APECED), and caspase recruitment domain-containing protein 9 (CARD9), IL-12R*β*1 and IL-12p40 deficiency, ROR*γ*T deficiency, STAT1 gain-of-function (2, 3). CMC's most common genetic cause is gain-of-function mutations in the STAT1 gene (STAT1-GOF); These mutations can lead to defective responses in Type 1 and Type 17 helper T cells (Th1 and Th17) (1). Besides being associated with fungal infections, GOF-STAT1 mutations are linked with autoimmune disorders such as vitiligo, thyroid disease, type I diabetes, psoriasis, and autoimmune hepatitis (AIH). Furthermore, CMC patients are more likely to develop aneurysms and malignancies. Patients with STAT1 functional mutations have a higher incidence of cerebral aneurysms than the healthy population, and CMC may be associated with the clinical manifestation of several primary immunodeficiency diseases (1, 2, 4, 5). As a result, AD STAT1 GOF is now categorized as syndromic CMC.

Here, we presented a CMC patient with liver dysfunction, oral candidiasis, acute bronchial pneumonia, and cytomegalovirus infection. It was a case of AD CMC with STAT1 mutation, and chronic active hepatitis may be related to this special situation.

## Case presentation

**Figure 1:**
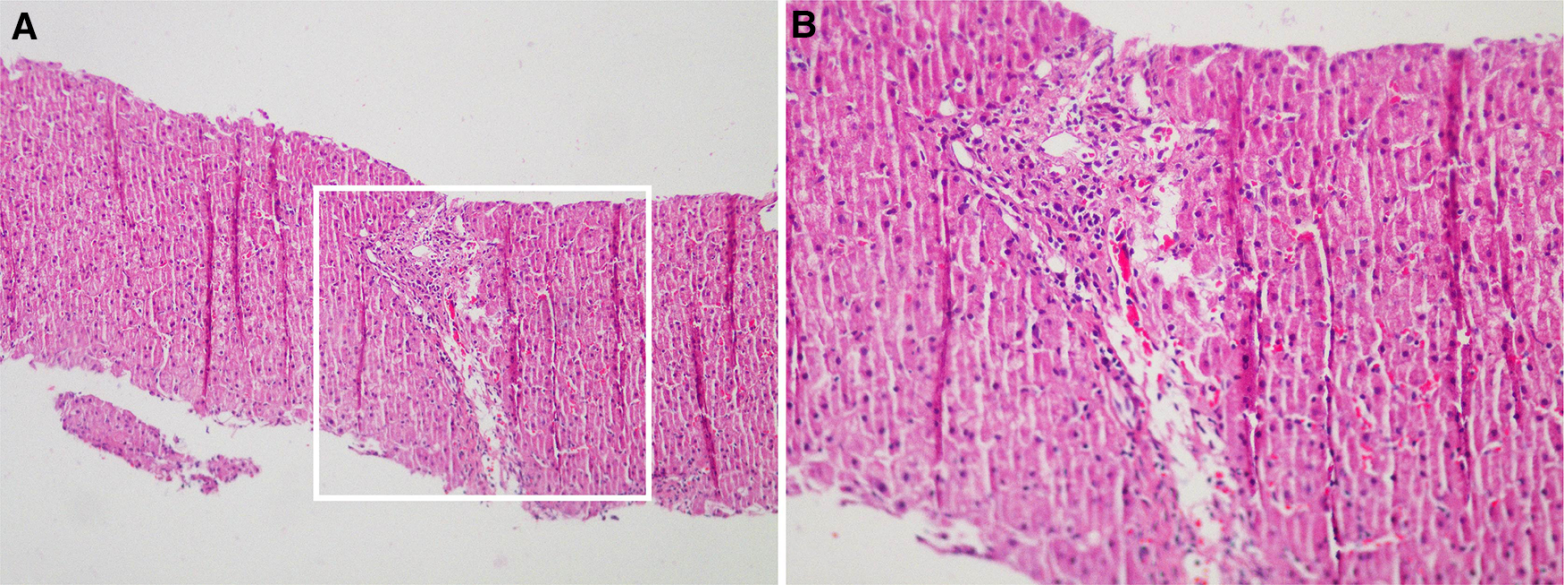
Liver perfusion analysis revealed incomplete lobular structure, partial hydrolysis of some hepatocytes, infiltration of a few lymphocytes into the portal area, and mild hyperplasia of the fibers. These observations were consistent with the pathological changes observed in G1S1 chronic hepatitis (**A**): Original magnification: 100×; (**B**): Original magnification: 200×).

**Figure 2:**
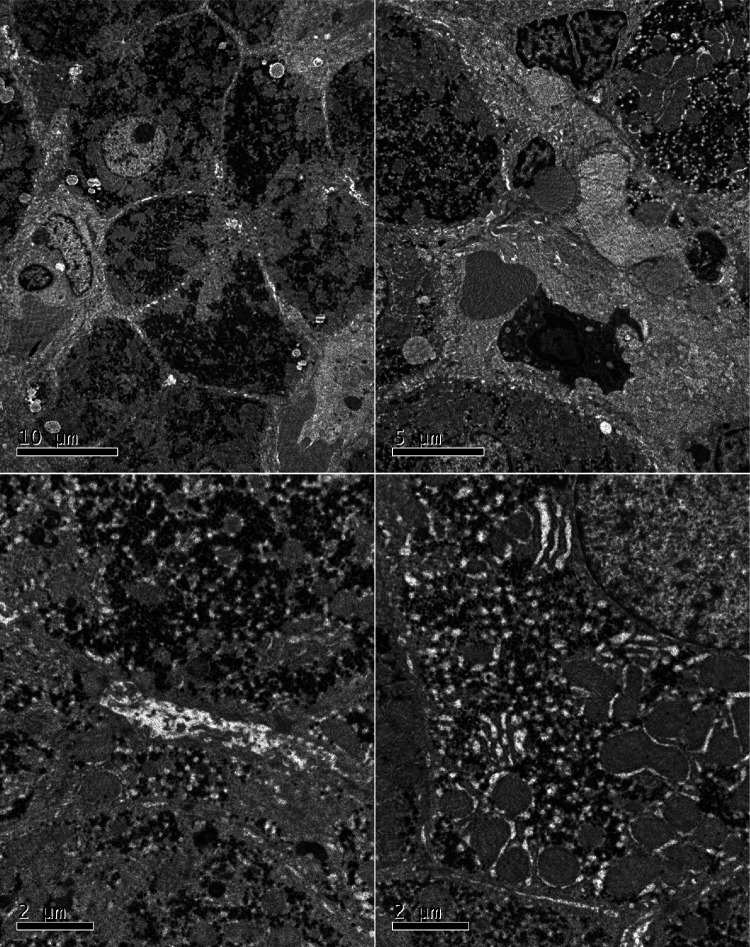
A liver biopsy under an electron microscope showed that the liver tissue was slightly swollen with a reduced endoplasmic reticulum, slight hyperplasia, expansion of the smooth endoplasmic reticulum, and blurred mitochondrial structures. Most of the hepatocytes showed a slight increase in the number of small lipid droplets in their cytoplasm, with few showing depositions of not many cholestatic pigment particles. The gaps between hepatocyte surfaces were slightly wider, and a few capillary bile ducts were somewhat dilated and cholestatic. Hepatic stellate cells were prominent in the Disse cavity, and focal bundles of collagen fibers were deposited. Kupffer cells were observed in the hepatic sinusoids with no portal area.

**Figure 3:**
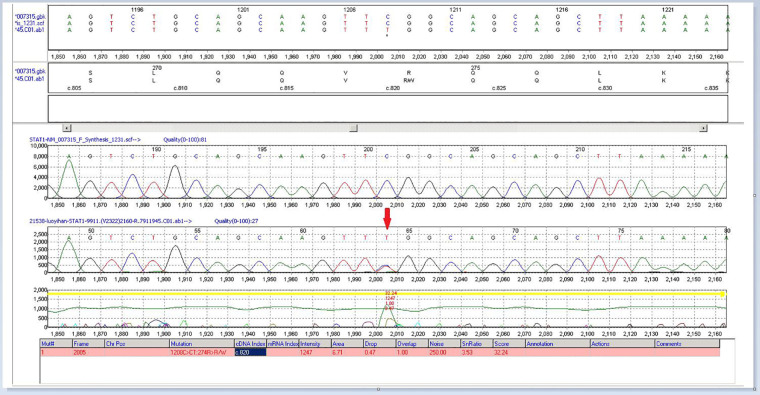
High-precision clinical display PLUS detection revealed a heterozygous GOF mutation (*p*.R274W) in the CC domain of the transcription factor STAT1.

A two-year-old girl was hospitalized for “hepatic dysfunction and recurring oral candidiasis for more than ten months.” She was hospitalized at a local hospital with “bronchitis” at the age of one year and one month, where she was found to have liver dysfunction with ALT and AST levels of 624 U/l and 644 U/l, respectively. Her condition improved after one week of treatment with glutathione and bicyclol. After continuing glutathione and bicyclol for more than one week, the liver enzyme levels returned to normal. At the age of one year and five months, she was treated at a local clinic for approximately three days due to oral candidiasis and poor appetite, and her ALT and AST levels were 1,019 U/L and 898 U/L respectively. She was admitted to the hospital. A liver, gallbladder, pancreas, and spleen ultrasound indicated gallbladder wall edema and splenomegaly. The child was then given anti-infective ceftezole and ganciclovir antiviral therapy. The treatment, however, proved ineffective, and the patient was moved to our hospital. Laboratory tests were performed during hospitalization with the following results: LDH 638 U/L (120–300 U/L), AST 1539 U/L (≤40 U/L), ALT 607 U/L (≤41 U/L), *γ*-GT 109 U/L (10–71 U/L), total cholesterol 2.52 mmol/L (<5.18), and total bile acid 58.3 µmol/L. There was no obvious coagulation abnormality. Screening for antibodies against hepatitis C virus (HCV), HAV, HEV, HIV, PVB, *Treponema pallidum* (TP), and hepatitis B virus (HBV) were negative; The concentration of Immunoglobulin A was 0.15 g/L (0.11–1.45 g/L), Immunoglobulin G was 8.8 g/L (3.3–12.3 g/L), Immunoglobulin M was 0.79 g/L (0.33–1.75 g/L), Complement C3 was 0.81 g/L (0.65–1.39 g/L), and Complement C4 was 0.23 g/L (0.16–0.38 g/L). The levels of blood ammonia (57 µmol/L) and ceruloplasmin (0.498 g/L), and the results of the lymphocyte subset analysis [T cells, B cells, and natural killer (NK) cells] indicated no significant abnormalities (Supplementary material Table S1). The level of anti-CMV-IgM and anti-CMV-IgG was 2.85 AU/ml and 89.74 AU/ml, respectively. IgG antibody against Epstein-Barr virus early antigen and nuclear antigen was <5.0 U/ml and 140.0 U/ml, respectively. The levels of IgG and IgM antibodies against Epstein-Barr virus capsid antigen were 39.2 U/ml and <10.0 U/ml, respectively. The Epstein-Barr virus DNA assay showed 9.08E + 002 copy/ml (0–500 copy/mL). *Mycobacterium tuberculosis* was not cultured either in a solid or liquid medium. The analysis of autoantibodies revealed cytoplasmic antinuclear antibodies as granule 1:100 and anti-liver cytosol-1 antibody (ALC-1) as suspicious, whereas screening for anti-GP210 antibody was weakly positive (Supplementary material Table S2). Retest The child was diagnosed with liver dysfunction, oral candidiasis, acute bronchial pneumonia, and cytomegalovirus infection. Liver protection was provided by administering enzyme-lowering (glutathione, bicyclol, magnesium isoglycyrrhizinate) and anti-infective (cefoperazone and sulbactam sodium, voriconazole) substances. Then the child was discharged and was orally administered bicyclol and glutathione tablets to reinforce treatment. For the next four months, liver function was measured in conventional outpatient clinics and was high (ALT: 50 U U/L, AST: 137 U/L). Since the cause for this elevated liver function remained unclear, the child was readmitted to the hospital. Liver biopsy and high-precision clinical display PLUS detection were conducted with the consent of the guardians. Liver perfusion analysis revealed incomplete lobular structure, partial hydrolysis of some hepatocytes, infiltration of a few lymphocytes into the portal area, and mild hyperplasia of the fibers. These observations were consistent with the pathological changes observed in G1S1 chronic hepatitis (Figure 1). A liver biopsy under an electron microscope showed that the liver tissue was slightly swollen with a reduced endoplasmic reticulum, slight hyperplasia, expansion of the smooth endoplasmic reticulum, and blurred mitochondrial structures. Most of the hepatocytes showed a slight increase in the number of small lipid droplets in their cytoplasm, with few showing depositions of not many cholestatic pigment particles. The gaps between hepatocyte surfaces were slightly wider, and a few capillary bile ducts were somewhat dilated and cholestatic. Hepatic stellate cells were prominent in the Disse cavity, and focal bundles of collagen fibers were deposited. Kupffer cells were observed in the hepatic sinusoids with no portal area (Figure 2). High-precision clinical display PLUS detection revealed a heterozygous GOF mutation (*p*.R274W) in the coiled-coil (CC) domain of the transcription factor STAT1 (Figure 3). The legal guardian provided written informed consent to participate in this study.

**Figure 4:**
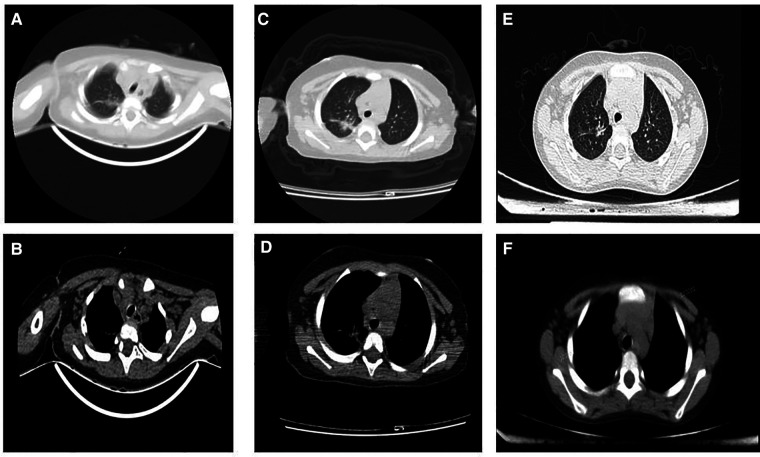
Serial radiologic investigations of the chest. (**A and B**): 2018.08.16 Infection in the upper middle lobe of the right lung, increased bilateral axillary lymph nodes. (**C** and **D**): 2019.01.17 Infection in the upper lobe of the right lung and lower lobe of both lungs, the lesions in the upper lobe of the right lung were slightly larger than before (2018-08-16); bilateral axillary lymph nodes increased. (**E** and **F**): 2021.08.16 The transparency of both lungs is uneven, considering the possibility of bronchiolitis, please combine the clinical; Mediastinal lymph node enlargement.

We monitored the clinical progress of this child for more than three years. After multiple treatments (antifungal voriconazole, nystatin treatment for oral Candida, antibiotics against bacterial infection, and liver protection with bicyclol and glutathione), the child still exhibited splenomegaly. Serial radiological investigations of the chest were conducted (Figure 4). We intermittently gave the child glutathione, bicyclol treatment, and oral antifungal therapy during this period. The child's family members were very cooperative with the doctor's treatment and brought her to the hospital regularly for re-examination to evaluate her liver function. Fortunately, the child's condition is stable, with no further clinical progress.

## Discussion

STAT1 gene mutation has been linked to several diseases. Inborn errors of human STAT1 immunity underlie 4 distinct disorders: autosomal recessive (AR) complete STAT1 deficiency, AR partial STAT1 deficiency, autosomal dominant (AD) STAT1 deficiency, and AD STAT1 gain-of-function. Each disease has its own set of clinical manifestations (6). STAT1 immunodeficiency type 31B with mycobacteria and viral infection is autosomal recessive, whereas the others are autosomal dominant. The STAT1 GOF phenotype is listed as Immunodeficiency 31C in the Online Mendelian Inheritance in Man and displays an autosomal-dominant inheritance pattern. These disorder clinical manifestations include primarily respiratory infections, immunodeficiency, and recurring infections with bacteria and other pathogens (5, 7, 8). STAT1 is the target of hereditary loss-of-function (LOF) or GOF mutations producing different clinical phenotypes. Patients with autosomal dominant STAT1- LOF mutations are infected with mycobacteria, whereas those with autosomal recessive STAT1 LOF mutations are prone to mycobacterial and viral infections, although the susceptibility to viral infection is not high. These mutations reflect the failure of interferon (IFN)-*γ* and IFN-α/*β*mediated immunity (4, 6, 9).

GOF-STAT1 mutations are associated with CMC, as reported by Liu et al. (4). STAT1 mutations strongly influence the pathogenesis of immunodeficiency diseases. Depner et al. (10) showed that GOF-STAT1 mutation attenuates the dephosphorylation of active STAT1 protein, leading to the accumulation of phosphorylated STAT1 in the nucleus. Sustained activation of STAT1 can alter the function of STAT1-dependent interleukin (IL)-17 inhibitors and inhibit the STAT3-induced differentiation of IL-17 T cells. GOF-STAT1 mutations lead to defects in the differentiation of Th17 T cells, which is characterized by decreased production of IL-17 and IL-22 (11). Since IL-17 and IL-22 play important antifungal roles in the skin and mucosa, GOF-STAT1 mutations might increase the susceptibility of CMC patients to fungal infection (12).

Using high-precision clinical display PLUS detection, genetic testing of the child revealed a heterozygous variation in the STAT1 gene: c.820C > T (*p*.R274W). Sequencing of the patient's mother and father simultaneously showed that this variation was inherited from the mother. The mutations affect the coiled-coil domain and impair the nuclear dephosphorylation that activates STAT1, which is related to their functional gain and advantage (4, 13, 14). Other harmful mutations at the same amino acid location (*p*.R274G, *p*.R274Q) have been reported in related clinical cases (5, 15). This mutation is not found in any population genetic database. The mutation-containing region of STAT1 is a key component whose amino acid sequence is highly conserved across species. According to the computer-aided study, this mutation most likely affects protein structure/function. The reports on the patient's clinical symptoms, the sequence analysis of her family, and the American College of Medical Genetics and Genomics (ACMG) published standards and guidelines for interpreting sequence variants (16) suggested that this variant was the cause of the disease. Besides developing chronic fungal infections, children can also develop chronic active hepatitis. Chronic active hepatitis is a rare complication with unknown pathogenesis. It might be related to viral infection, increased inflammatory cytokines and/or mutations (15–17). Clinical hepatitis caused by HCMV is relatively rare in immunocompromised populations; however, hepatitis is a well-known manifestation of HCMV infection in immunocompromised hosts, especially in liver transplant patients. The exact mechanism by which HCMV induces hepatitis is not fully established. People think that an inflammatory response with sustained cytokines (i.e., IL-17, IFN-*γ*, and TNF) release appear to be the dominant liver mechanism (17). Mutations in GOF-STAT1 lead to enhanced IFN-*γ*-induced gene expression but also impair the IFN-*γ* restimulation response (8). IFN-*γ* was higher in patients with various forms of fulminant hepatic failure. IFN-*γ*- strongly induces IL-18BP, which can buffer IL-18 activity in the liver through negative feedback. Excessive IL-18 immunity may be the pathogenic mechanism of fulminant liver failure (18, 19). Therefore, we speculate that viral infection and GOF-STAT1 gene mutation are involved in the pathogenesis of chronic hepatitis in children. In a study by Hori et al. a patient with chronic hepatitis also had a mutation in the same amino acid residue as that observed in our patient (*p*.R274Q) (16), indicating the contribution of specific STAT1 gene mutations (e.g., *p*.R274Q or *p*.R274W) to disease pathogenesis.

The clinical manifestations of CMC caused by GOF-STAT1 mutations are complex and range from mild local fungal infections to severe systemic diseases. Since CMC is an immunodeficiency disease, it is mainly treated with bone marrow transplantation, which allows the patients to develop cellular immunity against fungal infections, thus alleviating the clinical symptoms. Although bone marrow transplantation is not a routine treatment for CMC, it is effective, with successful cases reported in some studies (16, 20). However, bone marrow transplantation is limited by immune rejection. Additionally, since the relationship between genotype and clinical manifestation is not clear, determining the indications and best treatment options for bone marrow transplantation might be challenging. However, bone marrow transplantation might be the last resort for CMC patients receiving antifungal drugs with a poor prognosis, but further studies are needed to determine its effectiveness.

## Data Availability

The datasets presented in this study can be found in online repositories. The names of the repository/repositories and accession number(s) can be found in the article/**Supplementary Material**.
