# Supplementary material for: Autosomal dominant chronic mucocutaneous candidiasis with STAT1 mutation can be associated with chronic active hepatitis: A case report
Source: Front Pediatr. 2023 Jan 6;10:990729. doi: 10.3389/fped.2022.990729 (PMC9852885; doi:10.3389/fped.2022.990729)
Supplement: Supplementary file 1 [file Table1.docx]

Table 1 lymphocyte subset analysis

| **title** | **Outliers** | **Reference range** |
| --- | --- | --- |
| Total T lymphocytes (CD3+CD19-) (%) | 76.11% | 57.9-72.7 |
| NK cells (CD3-/CD16+CD56+) (#) | 641/ul | 90-900 |
| T lymphocytes + B lymphocytes + NK cells (%) | 99.18% | 95.00-105.00 |
| T lymphocytes + B lymphocytes + NK cells (#) | 6507/ul | - |
| Th/Ts | 2.01 | 1.33-2.77 |
| T lymphocytes (CD3+CD19-) (#) | 4994/ul | 700-4200 |
| Total B lymphocytes (CD3-CD19+) (%) | 13.30% | 15.4-28.3 |
| Total B lymphocytes (CD3-CD19+) (#) | 872/ul | 200-1600 |
| Helper/Induced T Lymphocytes (CD3+CD4+) (%) | 48.37% | 31.7-45.7 |
| Helper/inducing T lymphocytes (CD3+CD4+) (#) | 3174/ul | 300-2000 |
| Suppressive/cytotoxicT lymphocytes (CD3+CD8+) (%) | 24.09 % | 15.5-26.6 |
| Suppressive/cytotoxic T lymphocytes (CD3+CD8+) (#) | 1580/ul | 300-1800 |
| NK cells (CD3-/CD16+CD56+) (%) | 9.77% | 4.4-15.6 |
