# Supplementary material for: Autosomal dominant chronic mucocutaneous candidiasis with STAT1 mutation can be associated with chronic active hepatitis: A case report
Source: Front Pediatr. 2023 Jan 6;10:990729. doi: 10.3389/fped.2022.990729 (PMC9852885; doi:10.3389/fped.2022.990729)
Supplement: Supplementary file 2 [file Table2.docx]

Table 2 Autoantibodies

| title | result | Reference range |
| --- | --- | --- |
| Antinuclear antibody (ANA) | cytoplasmic granular type 1:100 | Negative (-); |
| Anti-smooth muscle antibody (ASMA) | (-) | Negative (-) positive (+) |
| Anti-liver-kidney microsomal-1 antibody (ALKM-1) | (-) | Negative (-) positive (+) |
| anti-mitochondrial-2  antibody | (-) | Negative (-) positive (+) |
| Anti-mitochondrial - M2-3E antibody | (-) | Negative (-) positive (+) |
| Anti -liver cytosol -1 antibody (ALC-1) | (±) | Negative (-) positive (+) |
| Anti-soluble liver antigen antibody | (-) | Negative (-) positive (+) |
| Anti-GP210 antibody | (±) | Negative (-) positive (+) |
| Anti-SP100 antibody | (-) | Negative (-) positive (+) |
| Anti-Ro-52 antibody | (-) | Negative (-) positive (+) |
